# Deciphering genetic mate choice: Not so simple in group‐housed conservation breeding programs

**DOI:** 10.1111/eva.12981

**Published:** 2020-05-19

**Authors:** Katherine A. Farquharson, Carolyn J. Hogg, Katherine Belov, Catherine E. Grueber

**Affiliations:** ^1^ School of Life and Environmental Sciences Faculty of Science The University of Sydney Sydney Australia; ^2^ San Diego Zoo Global San Diego USA

**Keywords:** captive breeding, DArTseq, genetic compatibility, genetic management, major histocompatibility complex, sexual selection

## Abstract

Incorporating mate choice into conservation breeding programs can improve reproduction and the retention of natural behaviors. However, different types of genetic‐based mate choice can have varied consequences for genetic diversity management. As a result, it is important to examine mechanisms of mate choice in captivity to assess its costs and benefits. Most research in this area has focused on experimental pairing trials; however, this resource‐intensive approach is not always feasible in captive settings and can interfere with other management constraints. We used generalized linear mixed models and permutation approaches to investigate overall breeding success in group‐housed Tasmanian devils at three nonmutually exclusive mate choice hypotheses: (a) advantage of heterozygous individuals, (b) advantage of dissimilar mates, and (c) optimum genetic distance, using both 1,948 genome‐wide SNPs and 12 MHC‐linked microsatellites. The managed devil insurance population is the largest such breeding program in Australia and is known to have high variance in reproductive success. We found that nongenetic factors such as age were the best predictors of breeding success in a competitive breeding scenario, with younger females and older males being more successful. We found no evidence of mate choice under the hypotheses tested. Mate choice varies among species and across environments, so we advocate for more studies in realistic captive management contexts as experimental or wild studies may not apply. Conservation managers must weigh up the need to wait for adequate sample sizes to detect mate choice with the risk that genetic changes may occur during this time in captivity. Our study shows that examining and integrating mate choice into the captive management of species housed in realistic, semi‐natural group‐based contexts may be more difficult than previously considered.

## 1 INTRODUCTION

Allowing for mate choice has long been suggested to improve the success of conservation breeding programs (Asa, Traylor‐Holzer, & Lacy, 2011; Martin‐Wintle, Wintle, Díez‐León, Swaisgood, & Asa, 2019; Quader, 2005; Schulte‐Hostedde & Mastromonaco, 2015; Wedekind, 2002). Any examination of the costs and benefits of allowing mate choice in a captive environment should reflect the long‐term demographic and genetic sustainability of the captive population (Chargé, Teplitsky, Sorci, & Low, 2014). For example, allowing mate choice may result in greater reproductive success overall and confer fitness benefits such as improved offspring health (see Martin‐Wintle et al., 2019 for a review in ex situ populations). Yet populations may also experience high reproductive skew if the individuals not preferred by others fail to breed. Reproductive skew can then result in loss of genetic diversity in small populations and a lower effective population size (Frankham, Ballou, & Briscoe, 2010). In their review of mate choice in captive management, Chargé et al. (2014) recognized that there is not sufficient theoretical and empirical evidence for guidelines that ensure fitness benefits without creating conflicts with other genetic goals. Therefore, an understanding of the mechanisms of mate choice in captivity is needed to ensure that overall genetic goals are not impeded. These goals often include benchmarks such as the maintenance of 95% genetic diversity over 100 years (Ballou et al., 2010) by equalizing genetic representation of wild‐born animals (founders) through preferentially breeding individuals with the lowest mean kinship (a measure of relatedness) in the population.

Much of the current literature on mate choice in conservation contexts focuses on experimental pairing trials. In pairing trials, an animal is housed with a test individual of the opposite sex, with behavioral indicators and/or reproductive outcomes used to determine whether the pairing is preferred or nonpreferred (e.g., Hartnett, Parrott, Mulder, Coulson, & Magrath, 2018; Martin‐Wintle et al., 2015; Parrott, Nation, & Selwood, 2019). Other studies compare the breeding success of pairings with varying genetic characteristics (Brandies, Grueber, Ivy, Hogg, & Belov, 2018; Parrott, Ward, & Temple‐Smith, 2006; Parrott, Ward, Temple‐Smith, & Selwood, 2015; Russell et al., 2018). While experimental trials are useful, they are labor‐intensive and require resources that may not be available in many conservation breeding programs; for example, the space required to house animals in pairs. Conservation breeding programs of threatened species may not be able to risk drops in productivity that could occur during experimental trials and forced monogamous pairings. Furthermore, for social species, housing individuals in pairs may not be conducive to normal behavioral expression (Lutz & Novak, 2005). As a result, there is a need to investigate mate choice hypotheses in observational studies using populations housed as they would be realistically managed in captivity, such as in group‐housed species.

The use of molecular markers in conservation breeding programs is increasingly common for a variety of management purposes, including resolving pedigrees, inferring population structure, and investigating hereditary diseases (Norman, Putnam, & Ivy, 2019). Molecular data gathered for these purposes can be extended to investigate mate choice. Evidence of mate choice may be found at the genomic level, which can be investigated using genome‐wide single nucleotide polymorphisms (SNPs) such as those generated with reduced representation sequencing (RRS) at a low cost. Mate choice may also be associated with variation in specific gene regions. For example, the involvement of the major histocompatibility complex (MHC) in disease resistance means that mate choice in relation to variation at this region may confer direct fitness benefits to offspring (Consuegra & Garcia de Leaniz, 2008). The MHC region has been widely linked to mate choice in a number of species, for a review of evidence see Kamiya, O'Dwyer, Westerdahl, Senior, and Nakagawa (2014).

A number of nonmutually exclusive genetic‐based mate choice hypotheses have been proposed, each with varying consequences for genetic goals of captive populations. In this study, we use both genome‐wide SNPs and MHC‐linked microsatellites to investigate the following three mate choice hypotheses in Tasmanian devils (*Sarcophilus harrisii*) housed in large free‐range enclosures. We use seven years of data from the largest managed captive breeding program in Australia (Hogg, Lee, Srb, & Hibbard, 2017), representing the best opportunity to detect mate choice without management intervention. The three hypotheses were the following:

1. Advantage of heterozygous individuals, where individuals with higher heterozygosity relative to those of the same sex show higher fitness (also known as quantity of alleles hypothesis; Doherty & Zinkernagel, 1975). If this occurs in a small population in captivity, individuals with lower heterozygosity will be less successful leading to reproductive skew, lower effective population size, and unequal founder representation at the population‐level over time. In a very small population, this may have the effect of changing allelic frequencies relative to the wild‐born founders, leading to genetic change over time. A benefit of our dataset, as opposed to wild studies, is that housing animals in known groups creates a discrete competitive mating environment, allowing us to test specific mate choice hypotheses such as the fitness benefits of heterozygosity. As distinct from the influence of absolute heterozygosity on overall breeding success, we predict the individuals with higher heterozygosity, relative to others of the same sex in the enclosure, to have a higher probability of successful reproduction regardless of their heterozygosity ranking relative to the larger population.
2. Advantage of dissimilar mates, where individuals that breed with mates most dissimilar to themselves can maximize heterozygosity and therefore fitness of their offspring (Landry, Garant, Duchesne, & Bernatchez, 2001), for example, by reducing inbreeding load (, 2007). As individuals will vary in their choice of mates, none should be disadvantaged, provided there is enough genetic diversity in the population to allow dissimilar pairings (Tregenza & Wedell, 2000). We predict that observed successful pairs will have greater dissimilarity than randomly selected pairwise combinations.
3. Optimum genetic distance, where individuals that breed with partners of an optimum level of genetic dissimilarity experience the greatest fitness. This hypothesis balances the potential effects of outbreeding depression due to breeding with too‐dissimilar mates, with inbreeding depression due to breeding with too‐similar mates (also related to the compatible genes hypothesis; Penn & Potts, 1999; Tregenza & Wedell, 2000). In a small population, genetic diversity will be depleted if the majority of successful pairings have high pairwise similarities (leading to inbreeding), or will increase with dissimilar pairings as per the advantage of dissimilar mates hypothesis (although outbreeding depression may be a potential risk; Chargé et al., 2014). Under this hypothesis, we predict that the variance in observed pairwise dissimilarities across enclosures will be lower than the variance in dissimilarity of random opposite‐sex pairs (e.g., Forsberg, Dannewitz, Petersson, & Grahn, 2007). This will occur if pairwise similarity converges on an optimum similarity value or tends toward this value if achieving the optimum distance was not possible for a particular grouping of individuals.

As with many captive programs, the Tasmanian devil insurance population is managed to meet conservation goals and so was not experimentally manipulated. Group‐housed devils exhibit high reproductive skew (approximately 60% of individuals fail to breed, Farquharson, Hogg, & Grueber, 2019), so an investigation of potential mate choice mechanisms driving this skew will inform ongoing management. By investigating mate choice hypotheses in a nonmanipulated captive setting, we aim to inform management options for other conservation breeding programs that house or plan to house species in groups with the opportunity for mate choice.

## 2 MATERIALS AND METHODS

### 2.1 Sampling

This study included 93 unique adult devils housed in two free‐range enclosures, Bridport and Freycinet, between 2011 and 2017. Free‐range enclosures are 22 ha in size and hold up to 21 adult devils in a roughly even sex ratio. Trapping within the free‐range enclosures occurs four times per year to monitor the health of devils and record breeding. Relative to one‐to‐one pairings on one extreme, and free‐roaming wild populations on the other extreme, the devil free‐range enclosures represent an intermediate level of management: offering a high potential for mate choice, while still under management (health checks and supplementary feeding; Grueber, Peel, Wright, Hogg, & Belov, 2018).

Some adults were present in more than one enclosure across the years, though none appeared in more than three enclosure years. An additional 15 devils that were housed at the sites during this time could not be included, as no DNA sample was obtained, or the sample was of too poor quality to sequence. A further five females were contracepted during some of the enclosure years for a separate study (Cope et al., 2018), none of which produced offspring. Contracepted devils were excluded from all analyses. A total of 123 offspring were observed in pouch checks, 34 of which did not survive to weaning (sampling) age so could not be included, and another four of which survived but were not sampled. Ear biopsies were collected by the Save the Tasmanian Devil Program under their Standard Operating Procedures for handling Tasmanian devils for management purposes, and DNA extracted using a phenol/chloroform protocol (Sambrook, Maniatis, & Fritsch, 1989). We considered a successful breeder as producing at least one offspring that survived until weaning, using the results of a molecular pedigree reconstruction performed with 891 SNPs and the R package "sequoia" (Huisman, 2017) to determine breeding status (Farquharson et al., 2019).

### 2.2 Nongenetic factors

We used data recorded in the Tasmanian devil studbook (Srb, 2018) and the ZIMS database (Species 360, 2018) to obtain the age and weight for every adult in each enclosure and year. Not all devils were trapped on each occasion, so we took the average weight of any records between 1st January and 30th April where the devil was held in that enclosure, as this time period covers the breeding season (Keeley et al., 2017). Average weight was reasonably consistent throughout the breeding season (female within‐individual mean coefficient of variation CV = 8.00% [min = 0%, max = 22.09%]; male within‐individual mean CV = 7.92% [min = 0.63%, max = 29.03%]). For one male that had no weight measurement, the closest measurement to this time (December of the previous year) was used.

### 2.3 Genome‐wide diversity

A reduced representation sequencing (RRS) approach was used to genotype genome‐wide SNPs by Diversity Arrays Technology Pty Ltd (DArTseq; Wenzl et al., 2004). To call and filter SNPs, we used a modified version of the Stacks pipeline (Catchen, Hohenlohe, Bassham, Amores, & Cresko, 2013) and a custom script written in R (R Core Team, 2018), as presented in Wright et al. (2019). We built a catalogue of 588 Tasmanian devil samples including those sequenced for this study and for other purposes, and filtered in Stacks on minimum genotyping rate (‐*r *.20), heterozygosity (‐‐max_obs_het 0.70), minor allele frequency (‐‐min_maf 0.01), and linkage equilibrium (‐‐write_random_snp). Within R, we further filtered on minimum average allelic depth (>2.5; to exclude loci with low allelic depth across the sample set at either the reference or alternate allele), coverage difference (<80%), reproducibility between technical replicates (>90%), and minor allele frequency (>5%) to obtain 1,948 SNPs across the samples relevant to this analysis to calculate diversity metrics.

We chose standardized genome‐wide heterozygosity (*H*_GW_) as our measure of genome‐wide diversity, calculated as the total number of heterozygous loci in a sample divided by the sum of the average observed heterozygosities for all samples at the same genotyped loci, using the "inbreedR" package in R (Stoffel et al., 2016). A standardized metric reduces the influence of missing data.

### 2.4 MHC diversity

We typed the adults (48 males, 43 females) for which we had sufficient DNA at 12 MHC‐linked microsatellite loci (Table S1) developed by Cheng and Belov (2014) and Day et al. (2019). Polymerase chain reactions (PCRs) with Qiagen Type‐It Microsatellite PCR Kit were performed in a 10 μl reaction with 1 μl of ~12 ng/μl template DNA, and 0.2 μM of the forward and reverse primer for each locus. Amplification of PCR products was performed on a T100 Thermal Cycler (Bio‐Rad) with a 5 min 95°C enzyme activation step, followed by 30 cycles of 30 s at 95°C denaturation, 90 s annealing at 65°C, and 30 s extension at 72°C, before a final 30 min extension at 60°C. Capillary electrophoresis on an Abi 3130XL Genetic Analyzer (Applied Biosystems) separated fragments for allele scoring using GeneMarker 1.95 (Soft Genetics LLC) against the McLab DMSO 100 size standard (Molecular Cloning Laboratories).

Similar to *H*_GW_, we standardized MHC‐based heterozygosity (*H*_MHC_) for each individual. Our two measures of genetic diversity, *H*_MHC_ and *H*_GW_, were weakly correlated across the dataset (*r* = −.25 in females, *r *= −.14 in males; *r* = −.21 in unique females, *r* = −.09 in unique males; Figure S1), as were all other input variables (age and weight correlations < 0.3).

### 2.5 Overall breeding success

We first investigated the factors affecting breeding success (production of an offspring that survived until weaning) using our entire dataset. Modeling both sexes together would require multiple interaction terms to be fitted to account for age and weight differences between the sexes, which was not feasible given our sample sizes. Males and females were therefore analyzed in separate models containing age, average weight, and the two standardized genetic diversity metrics, *H*_GW_ and *H*_MHC_, as fixed predictors. Ideally, we would include the random effects of enclosure, year, and individual ID to account for variation in breeding success among the two free‐range enclosures, multiple years, and repeated breeding attempts of some individuals. However, some of these could not be fitted due to convergence issues, likely due to low variance and sample size constraints; for example, 24 of 44 unique females appeared only once in the dataset with no female appearing more than three times. Therefore, we only fitted random intercepts with adequate variation to avoid convergence issues for each model, being the individual ID for males:

$$\text{Breeding Success}\;∼\;\text{Age}\;+\;\text{Average weight}\;+\;H_{\text{GW}}\;+\;H_{\text{MHC}}\;+\;\left(1|\text{ID}\right),$$

and the enclosure year for females:

$$\text{Breeding Success}\;∼\;\text{Age}\;+\;\text{Average weight}\;+\;H_{\text{GW}}\;+\;H_{\text{MHC}}\;+\;\left(1|\text{EnclosureYear}\right)$$

Generalized linear mixed models, with a binomial response for successful (1) or unsuccessful (0) breeders, were estimated with the "glmer" function from the "lme4" package in R. Model averaging and model selection using an information theoretic approach following Grueber, Nakagawa, Laws, and Jamieson (2011) were used to obtain the most adequate model. Briefly, input variables were standardized to improve model inference (effects on same scale for direct comparison of magnitude) by dividing by 2 *SD* following Gelman (2008). Sub‐models of the global model (containing all parameters of interest) were obtained using the "MuMIn" package (Barton, 2018), and models within the top 2 AICc of the best model were averaged using the full average method. Details of the top model sets are provided in Table S2, along with conditional *R* ^2^ values (Nakagawa & Schielzeth, 2013). Estimates with a relative importance (RI) of 1 (indicating the parameter was included in all top model sets) were back‐transformed for interpretation.

Additionally, for successful breeders, we modeled the number of offspring produced using the same four factors of interest. As this reduced the sample size, for males a generalized linear model was used (only three males were repeated so ID could not be fit as a random effect), with a Poisson error distribution. For females that are biologically limited to producing a maximum of four offspring, a two‐column vector of successes (number of offspring) and failures (4—number of offspring) was used as the binomial response.

### 2.6 Hypothesis 1: advantage of heterozygous individuals

We tested relative effects (e.g., whether more heterozygous males in an enclosure were more successful than less heterozygous males in that enclosure, regardless of their heterozygosity in comparison with all genotyped males in all enclosures) by standardizing all four predictors, age, weight, *H*_GW,_ and *H*_MHC_ within each enclosure and sex. Predictors were standardized by calculating the difference from the group mean and dividing by 2 *SD* of the group values; male and female models were rerun as above for both breeding success and number of offspring responses. Models were specified as above, but using the relative measures of heterozygosity, weight, and age instead of absolute measures.

### 2.7 Hypothesis 2: advantage of dissimilar mates

To test the hypothesis that breeders are most successful when they pair with dissimilar mates that maximize heterozygosity of their offspring (relative to a random mate selection), we calculated pairwise genetic similarity as $D_{\text{AB}}\;=\;2\;\times \;F_{\text{AB}}/\left(F_{A}\;+\;F_{B}\right)$ , where *F*_A_ is the total alleles of female A, *F*_B_ is the total alleles of male B, and *F*_AB_ is the total number of unique alleles shared by female A and male B (Wetton, Carter, Parkin, & Walters, 1987). Similarity was calculated between every possible opposite‐sex pairing for each enclosure, and separately at the genome‐wide SNPs and the MHC loci for which both individuals of the pair were sequenced. For each enclosure, we then compared the average pairwise similarity of the observed successful breeding pairs to an expected average. The expected average was calculated from a structured randomization simulation, written in R, that selected the same number of pairings as were observed to breed from the set of possible pairings for that enclosure (with equal sex ratios as some males bred with multiple females and vice versa). The simulation was repeated 100,000 times to ensure adequate parameter space exploration. As some years had small numbers of observed successful pairings, we also pooled all enclosure years to obtain an overall estimate of observed versus expected mean pairwise similarities using the structured simulation, modified to account for the additional structure due to enclosure year group. We interpret evidence of advantage of dissimilar mates as an observed mean lower than the 95% confidence interval of the expected mean similarity under random mating.

### 2.8 Hypothesis 3: optimum genetic distance

Observed pairwise genetic similarities below the expected range (i.e., successful pairs were more different from one another than expected under random mating) may indicate increased fitness of dissimilar mates. In contrast, observing a high proportion of pairwise similarities falling within the expected range is also predicted under the optimum genetic distance hypothesis, if the optimum heterozygosity is close to the mean heterozygosity. If animals that mate with individuals at an optimal genetic distance to themselves are more likely to successfully breed than other pairings, we would expect the standard deviation of the observed pairwise dissimilarities to be lower than that of the expected standard deviation of successful pairings that would occur under random mating. We therefore compared the observed standard deviation in pairwise similarities of the observed successful breeders to the standard deviation under simulated random mating, similar to the approach of Forsberg et al. (2007) and Lenz, Hafer, Samonte, Yeates, & Milinski, 2018.

## 3 RESULTS

### 3.1 Overall breeding success

Overall breeding success of females across our dataset had a negative relationship with age (61% probability of breeding success at age 2 versus 47% at age 3, 34% at age 4, and 22% at age 5, fitted values from model presented in Table 1). Average weight was excluded from the final model for females but had a positive relationship with overall breeding success for males (RI = 1, Table 1). The two genetic predictors, *H*_GW_ and *H*_MHC_, had low model selection certainty (RI < 1) as predictors of overall breeding success for both females and males. Examining successful breeders only, average weight had a negative relationship with the number of offspring produced in females, but there were no strong predictors for males (Table S3).

**TABLE 1:** Overall breeding success results for females and males after model averaging

	Predictor	Estimate^a^ (unconditional SE)	RI^b^
Females (*N* = 74)	Intercept	−0.1529 (0.3064)	
	Age	−1.0503 (0.5526)	1
	*H*_GW_ ^c^	0.1097 (0.3427)	0.25
	*H*_MHC_ ^c^	0.0567 (0.2699)	0.20
Males (*N* = 69)	Intercept	−0.6325 (0.3783)	
	Age	0.1886 (0.5079)	0.23
	Average weight	1.6426 (1.0120)	1
	*H*_GW_ ^c^	1.1710 (1.0991)	0.78
	*H*_MHC_ ^c^	−0.1011 (0.3748)	0.17

Note

Breeding success (1 = success, 0 = failure) was the binomial response variable.

^a^ Estimates have been standardized on 2 *SD* following Gelman (2008).

^b^ RI is the relative importance of the predictor in the final model, calculated as the proportion of top models the predictor was included in.

^c^ Genome‐wide heterozygosity (*H*_GW_) and MHC heterozygosity (*H*_MHC_) were standardized across all loci for which an individual was genotyped to reduce the influence of missing data on the analysis.

### 3.2 Advantage of heterozygous individuals

We found no evidence that males or females with high genome‐wide or MHC‐linked heterozygosity, relative to others in the enclosure, had greater breeding success (Table 2). *H*_MHC_ was not included in any of our final models, while *H*_GW_ had a low relative importance (low model selection certainty). Age showed a negative relationship with breeding success for females in competitive environments as per the overall breeding success model. The youngest female relative to the average age of the other females in the enclosure had a 73% fitted probability of breeding success compared to 17% for the oldest female in the enclosure. Conversely, age had a positive relationship with breeding success for males in competitive environments. The youngest male relative to the average age of the other males in the enclosure had a 17% fitted probability of breeding success compared to 65% in the oldest male. However, absolute age was not an important predictor for overall male breeding success (Table 1). Similar to the overall breeding success models, average weight was negatively related to the number of offspring produced in females, but there were no strong predictors of number of offspring produced in males (Table S4).

**TABLE 2:** Results of “advantage of heterozygous individuals” hypothesis tested in a competitive breeding scenario

	Predictor^a^	Estimate^b^ (unconditional SE)	RI^c^
Females (*N* = 74)	Intercept	−0.1576 (0.3088)	
	z.Age	−1.0744 (0.5194)	1
	z.Average weight	−0.1101 (0.3215)	0.25
	z.*H*_GW_ ^d^	0.0606 (0.2615)	0.21
Males (*N* = 69)	Intercept	−0.5914 (0.3293)	
	z.Age	1.3078 (0.7160)	1
	z.*H*_GW_ ^d^	0.5462 (0.7263)	0.54

Note

Breeding success (1 = success, 0 = failure) was the binomial response variable.

^a^ All predictors were converted to z‐scores within each enclosure year and sex before input to models to reflect competition among individuals.

^b^ Estimates have been standardized on 2 *SD* following Gelman (2008).

^c^ RI is the relative importance of the predictor in the final model, calculated as the proportion of top models the predictor was included in.

^d^ Genome‐wide heterozygosity (*H*_GW_) was standardized across all loci for which an individual was genotyped to reduce the influence of missing data on the analysis.

### 3.3 Advantage of dissimilar mates

**FIGURE 1:**
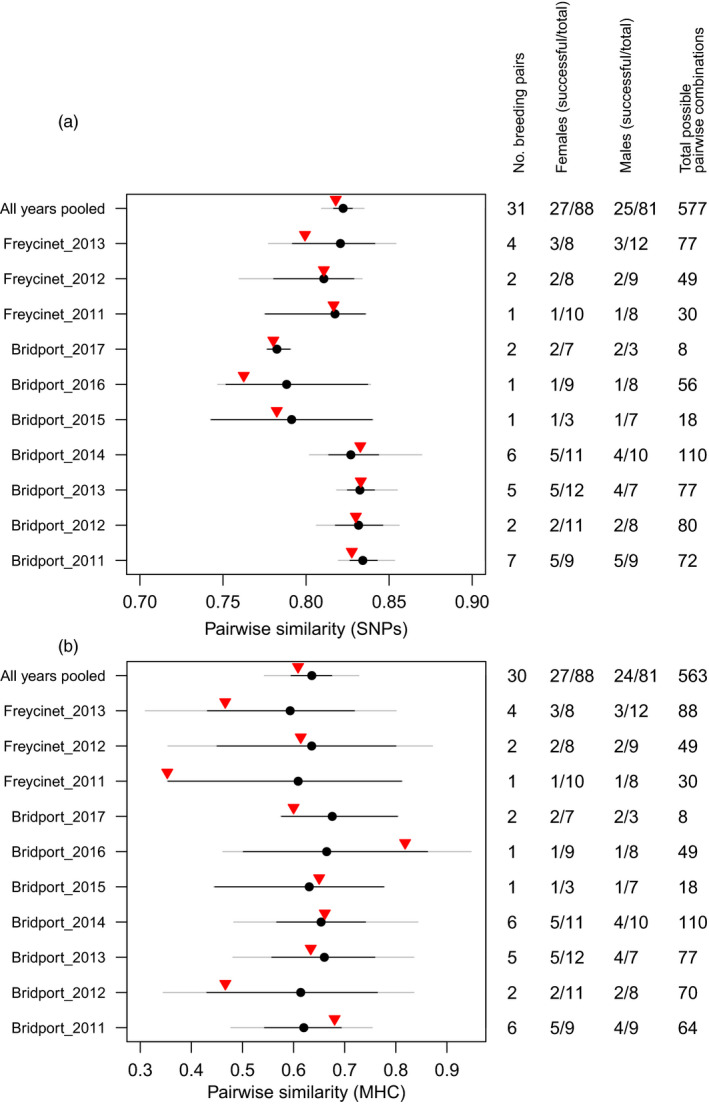
Expected versus observed similarity for advantage of dissimilar mates hypothesis. Observed mean pairwise similarity of successful breeding pairs (red triangle) versus expected mean pairwise similarity (black circle) of 100,000 simulated pairings under the same conditions with 95% CIs, calculated from (a) genome‐wide SNP data and (b) MHC‐linked microsatellite loci. Minimum and maximum simulated values outside of 95% CI shown by gray line

For both genome‐wide SNPs and MHC‐linked microsatellites, observed mean pairwise similarities for each enclosure and year fell within the 95% CI for the expected mean under random mating (Figure 1a,b). We observed no patterns across years or enclosures (i.e., observed values were not consistently below or above the expected mean), providing no evidence to support the advantage of dissimilar mates hypothesis (Figure 1a,b).

### 3.4 Optimum genetic distance

**FIGURE 2:**
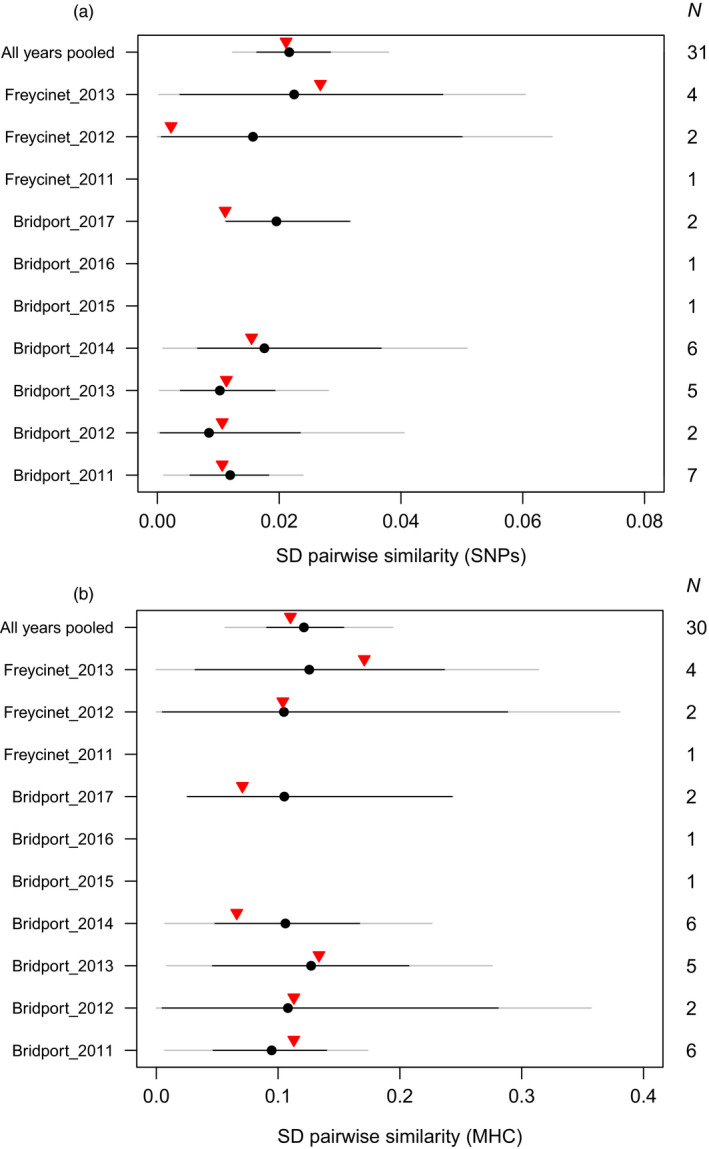
Optimum genetic distance hypothesis. Observed standard deviation (*SD*) in pairwise similarity of successful breeding pairs (red triangle) versus mean expected standard deviation in pairwise similarity (black circle) of 100,000 simulated pairings under the same conditions with 95% CIs (black lines), calculated from (a) genome‐wide SNP data and (b) MHC‐linked microsatellite loci. *N* is the number of successful breeding pairs for which sequence data were available in each enclosure year (see Figure 1 for more detail). Note that enclosure years with a sample size of one successful pair were excluded as no standard deviation could be calculated. Minimum and maximum simulated values outside of 95% CI shown by gray line

The standard deviation of pairwise similarities among successful breeders was close to the expected value under random mating (within the 95% confidence interval) for both genome‐wide SNPs and MHC‐linked microsatellites (Figure 2a,b), providing no evidence to support the optimum genetic distance hypothesis.

## 4 DISCUSSION

Mate choice is often touted as a reason to group‐house individuals in captivity (Wedekind, 2002), yet is rarely tested in realistic group‐housing scenarios. As a result, the impact of mate choice on conservation breeding programs that utilize group‐housing is not clearly understood. Here, we used a large observational dataset of captive group‐housed Tasmanian devils to test three mate choice hypotheses. We found no evidence to support any of these genetic mate choice processes using either MHC‐linked microsatellite loci or genome‐wide SNP loci. Therefore it is possible that none of these hypotheses account for the high reproductive skew observed in this population (Farquharson et al., 2019), or that we did not have the power to detect weak effects. Whilst we accounted for individual replicates in males, our female model included pseudoreplicates as only the “EnclosureYear” random effect could be fitted. “EnclosureYear” does itself account for temporal pseudoreplication but not at the level of the individual. Pseudoreplication underestimates variation, increasing the probability of detecting a significant result when there is none (Type I error). As we did not detect any significant effects at either MHC‐linked or genome‐wide measures, we believe our conclusions are robust to pseudoreplication. It is possible however, that the significant female age finding was inflated by pseudoreplication. Nevertheless, the negative influence of age on breeding success is in line with other studies within the same species (Farquharson, Hogg, & Grueber, 2017; Russell et al., 2018).

Selection coefficients for diversity‐ and dissimilarity‐based mate choice processes are likely to be weak (Kamiya et al., 2014), meaning that a large amount of data would be needed to detect any trend. The range of possible expected values exhibited under our random mating simulations was great enough to potentially detect observed values outside of the 95% CI in approximately 7 of the 10 enclosure years that we examined (gray bars Figure 1), also demonstrating that our captive study population has enough genetic diversity to generate dissimilar pairings. For the other three enclosure years, the 95% CI covered the full range of possible values. Nevertheless, we did not detect conclusive deviations from random mating, even when data from all years were pooled together. As the observed effects did not follow a pattern (i.e., did not all trend below or above the expected mean), we consider it unlikely that we would detect any pattern even with increased sample sizes.

The MHC is widely used in mate choice studies, yet it is likely that other genomic regions are also involved in mate choice and/or reproductive success. One such example is secondary sexual characteristics that may be reliable indicators of general mate quality (Møller & Alatalo, 1999). These secondary sexual characteristics may also be associated with MHC diversity, such as in white‐tailed deer where the development of antlers is associated with allelic diversity at the MHC‐*DRB* gene (Ditchkoff, Lochmiller, Masters, Hoofer, & Bussche, 2001). Our study utilized SNP and MHC‐linked microsatellite data. Microsatellite‐linked markers may underestimate preferences for functional MHC diversity as a result of linkage equilibrium. Therefore, gene sequence data would be needed to determine underlying mechanisms. Tasmanian devils do not display any known secondary sexual characteristics, and although males are slightly larger than females, the species is not clearly sexually dimorphic. We did note however that the older males and younger females tend to have higher reproductive success (Table 2). While no genetic factors influenced breeding success in our population, age was important for both males and females in competitive breeding environments. Age was also important for the overall breeding success of females, but not for the overall breeding success of males where average weight was strongly positively correlated with breeding success. This suggests that in enclosures with a range of ages, relatively older males may be able to dominate breeding events, though the absolute oldest males may not be the most successful overall. Similar results have been found in male fallow deer (*Dama dama*), where dominance rank was positively correlated with age but there was no age effect on reproductive behavior after controlling for dominance (Komers, Pélabon, & Stenström, 1997). In deer, younger males may successfully reproduce in low competition scenarios, but reduce their mating behaviors when competition increases (Komers et al., 1997). Behavioral studies of enclosure use and social interactions will assist in uncovering subtle age‐related effects. Captive managers aiming to breed from young animals should therefore consider limiting the number of older males housed in the same enclosure for a higher success.

We can compare our findings here to results of devil studies under other housing conditions, to determine how the influence of nongenetic factors may vary between environments, even within a species. In smaller captive enclosures (up to four males, as opposed to up to 11 males herein), Gooley, Hogg, Belov, and Grueber (2018) found that weight influenced male breeding success, similar to our findings for overall male breeding success (Table 1). However, weight was excluded from our final competitive breeding models, so it may be less important in competitive scenarios. An explanation for this difference may be that in smaller enclosures, heavier (i.e., larger) males are able to dominate breeding by mate guarding, a known behavior in devils (Guiler, 1970), while in larger enclosures, the increased male competition may reduce the advantage of weight. Large free‐range enclosures with a greater number of adults will limit the ability of dominant males to guard all reproductive females. Compared to studies of devils housed in one‐on‐one pairs without opportunity for mate choice, we found similar effects of female age on reproductive success (Farquharson et al., 2017; Russell et al., 2018).

A genetic study by Russell et al. (2018) found that devil pairs with different numbers of heterozygous loci had a higher probability of breeding success than pairs with similar heterozygosities, using 6 of the MHC‐linked microsatellites that were also included in our study. On the other hand, Day et al. (2019) did not detect mate choice using MHC‐linked microsatellites in smaller group enclosures when examining overall MHC heterozygosity. Both of these studies also accounted for age. Taken together with the results of the current study, we can infer that detection of mate choice varies across captive environment types and that perhaps group size may be an important driver of mate choice expression and/or competition. While genetic‐based mate choice may influence the reproductive success of forced monogamous pairings that do not experience competition, nongenetic factors contributing to behavioral dominance such as age and weight come into play in mating competition and could mask any influence of MHC‐associated reproductive success. Experimental mate choice trials typically do not consider factors such as density and competition, so may be providing unrealistic estimates of the importance of heterozygosity or genetic dissimilarity in captive populations as a consequence of their sampling design.

Several authors have called for empirical studies of mate choice in conservation breeding programs (Asa et al., 2011; Chargé et al., 2014). By examining the largest managed captive breeding program in Australia, we had a unique opportunity to detect mate choice in a management context without experimental intervention. We did not find any evidence that devil breeding success was driven by any of the mate choice hypotheses we tested. It is possible that mate choice is occurring, either via an untested mechanism or via the mechanisms we tested but with an effect size that is too weak for us to detect in this population. In general, the effect of heterozygosity on fitness is typically weak (Chapman, Nakagawa, Coltman, Slate, & Sheldon, 2009; Szulkin, Bierne, & David, 2010). However, the influence of heterozygosity at specific gene regions such as the MHC is expected to be stronger than the genome‐wide average (Hedrick, 2012). If it is true that underlying effect sizes are weak in our study system, it is difficult to conceive management strategies that could be informed by this process to improve progress toward conservation genetic goals. It is also possible that breeding success in devils is influenced by unmeasured traits, as our study population exhibits a high reproductive skew, with almost two‐thirds of individuals failing to breed given an opportunity in free‐range enclosures (Farquharson et al., 2019). Importantly, although reproductive skew decreases effective population size overall (Frankham et al., 2010), our current study shows that allowing mate choice by housing devils in groups does not appear to exacerbate genetic change at the MHC as all observed values were within the expected range under random mating.

Although experimental studies promote the use of group‐housing to provide mate choice, the potential costs in respect of genetic diversity may be high (Chargé et al., 2014). The strength and type of mate choice are not necessarily fixed within species, and can vary based on environmental (e.g., Robinson, Sander van Doorn, Gustafsson, & Qvarnström, 2012) or social conditions (such as population density, for example, Martinossi‐Allibert, Rueffler, Arnqvist, & Berger, 2019; Sharp & Agrawal, 2008). This is likely true for devils, as inferences vary across contexts (e.g., Day et al., 2019; Farquharson et al., 2017; Gooley et al., 2018; Russell et al., 2018; see above). Managers are already aware of the need to collect and genotype samples for all individuals in realistic contexts to accurately assign breeding outcomes. A remaining challenge for conservation managers will be balancing the time taken to obtain sufficient sample sizes to detect any (possibly weak) effect, with the risk that mate choice may influence the genetic structure of the population during that time. For conservation management to be informed by mate choice theory, we advocate for more studies in realistic captive management contexts, as opposed to solely experimental or wild studies, which may not apply.

## CONFLICT OF INTEREST

None declared.

### DATA ACCESSIBILITY

The data and code underlying this study are available from the public database Dryad (https://doi.org/10.5061/dryad.zs7h44j5q) (Farquharson, Hogg, Belov, & Grueber, 2020). Data include individual genotypes at the 1,948 SNPs and 12 MHC‐linked microsatellites, and weight, age, and enclosure information.

## Supporting information

Supplementary Material
