# Supplementary material for: Deciphering genetic mate choice: Not so simple in group‐housed conservation breeding programs
Source: Evol Appl. 2020 May 19;13(9):2179–89. doi: 10.1111/eva.12981 (PMC7513713; doi:10.1111/eva.12981)
Supplement: Supplementary file 1 — Supplementary Material [file EVA-13-2179-s001.docx]

Supplementary Table 1: Characteristics of 12 MHC-linked microsatellites.

All markers developed by Cheng and Belov (2014), with the exception of MHCI12 (Day *et al.*, 2019).

| Locus | MHC class linked | Multiplex | Fluorescent tag |
| --- | --- | --- | --- |
| MHCI06 | I | MHC 1 | NED |
| MHCI09 | I | MHC 1 | 6-FAM |
| MHCI08 | I | MHC 1 | PET |
| MHCI07 | I | MHC 2 | NED |
| MHCI02 | I | MHC 2 | PET |
| MHCI10 | I | MHC 2 | NED |
| MHCII02 | II | MHC 2 | VIC |
| MHCI05 | I | MHC 3 | 6-FAM |
| MHCI11 | II | MHC 3 | VIC |
| MHCII03 | II | MHC 3 | PET |
| MHCI01 | I | MHC 3 | NED |
| MHCI12 | I | N/A | 6-FAM |

Supplementary Table 2: Top model set (top 2 AIC_C_) of generalised linear mixed models for overall breeding success and relative breeding success (standardised across competitive breeding enclosure).

Female models were fitted with a random intercept for “enclosure.year”, male models were fitted with a random intercept for “ID”. The final models are provided in Table 1 and 2.

| Model statement | AIC_C_ | Δ*_i_*^a^ | *w_i_*^b^ | R^2 c^ |
| --- | --- | --- | --- | --- |
| *Females (overall)* |  |  |  | 0.158 |
| β_0_ + Age | 103.8 |  | 0.262 | 0.141 |
| β_0_ + Age + *H*_GW_^d^ | 105.4 | 1.59 | 0.118 | 0.155 |
| β_0_ + Age + *H*_MHC_^d^ | 105.8 | 1.98 | 0.097 | 0.142 |
| *Males (overall)* |  |  |  | 0.477 |
| β_0_ + Average weight + *H*_GW_^d^ | 92.0 |  | 0.191 | 0.445 |
| β_0_ + Age + Average weight + *H*_GW_^d^ | 93.0 | 1.02 | 0.115 | 0.482 |
| β_0_ + Average weight | 93.1 | 1.08 | 0.111 | 0.341 |
| β_0_ + Average weight + *H*_GW_^d^ + *H*_MHC_^d^ | 93.6 | 1.61 | 0.085 | 0.437 |
| *Females (relative)* ^e^ |  |  |  | 0.170 |
| β_0_ + z.Age | 103.3 |  | 0.275 | 0.147 |
| β_0_ + z.Age + z.Average weight | 104.8 | 1.50 | 0.130 | 0.161 |
| β_0_ + z.Age + z.*H*_GW_^d^ | 105.2 | 1.90 | 0.106 | 0.154 |
| *Males (relative)* ^e^ |  |  |  | 0.313 |
| β_0_ + z.Age + z.*H*_GW_^d^ | 92.7 |  | 0.214 | 0.301 |
| β_0_ + z.Age | 93.0 | 0.30 | 0.184 | 0.247 |

^a^ Change in AIC_C_ from the best model.

^b^ Akaike model weight.

^c^ Conditional R^2^ calculated for global model, and then separately for each of the submodels in the top model set.

^d^ Genome-wide heterozygosity (*H*_GW_) and MHC heterozygosity (*H*_MHC_) were standardised across all loci for which an individual was genotyped to reduce the influence of missing data on the analysis.

^e^ All predictors were converted to z-scores within each enclosure year and sex before input to models to reflect competition amongst individuals.

Supplementary Table 3: Number of offspring results for females and males after model averaging.

Female models were fitted as a two-column binomial response of success/failures (as they are biologically limited to a maximum of four offspring). Male models were fitted with a Poisson distribution. Only successful breeders were included in the models. Details of the top model sets are provided in Supplementary Table 5.

|  | Predictor | Estimate^a^ (unconditional SE) | RI^b^ |
| --- | --- | --- | --- |
| Females (*N* = 34) | Intercept | 0.3356 (0.2626) |  |
|  | Average weight | -1.0223 (0.4688) | 1 |
|  | *H*_MHC_^c^ | 0.4933 (0.5654) | 0.57 |
|  | *H*_GW_^c^ | -0.1821 (0.3682) | 0.35 |
| Males (*N* = 26) | Intercept | 0.9903 (0.1272) |  |
|  | Average weight | 0.1331 (0.2247) | 0.38 |
|  | *H*_MHC_^c^ | -0.1107 (0.2187) | 0.33 |
|  | Age | -0.0657 (0.1724) | 0.22 |
|  | *H*_GW_^c^ | -0.0672 (0.1831) | 0.20 |

^a^ Estimates have been standardised on 2 SD following Gelman (2008).

^b^ RI is the relative importance of the predictor in the final model, calculated as the proportion of top models the predictor was included in.

^c^ Genome-wide heterozygosity (*H*_GW_) and MHC heterozygosity (*H*_MHC_) were standardised across all loci for which an individual was genotyped to reduce the influence of missing data on the analysis.

Supplementary Table 4: Results of “advantage of heterozygous individuals” hypothesis tested in a competitive breeding scenario, using number of offspring as the response.

Female models were fitted as a two-column binomial response of success/failures (as they are biologically limited to a maximum of four offspring). Male models were fitted with a Poisson distribution. Only successful breeders were included in the models. Details of the top model sets are provided in Supplementary Table 5.

|  | Predictor^a^ | Estimate^b^ (unconditional SE) | RI^c^ |
| --- | --- | --- | --- |
| Females (*N* = 34) | Intercept | 0.3787 (0.2323) |  |
|  | z.Average weight | -1.3295 (0.4301) | 1 |
|  | z.*H*_MHC_^d^ | 0.0972 (0.2650) | 0.29 |
| Males (*N* = 26) | Intercept | 0.9803 (0.1288) |  |
|  | z.*H*_GW_^d^ | -0.3374 (0.3062) | 0.72 |
|  | z.Average weight | 0.1049 (0.2057) | 0.34 |
|  | z.*H*_MHC_^d^ | -0.0916 (0.2063) | 0.25 |
|  | z.Age | -0.0379 (0.1372) | 0.12 |

^a^ All predictors were converted to z-scores within each enclosure year and sex before input to models to reflect competition amongst individuals.

^b^ Estimates have been standardised on 2 SD following Gelman (2008).

^c^ RI is the relative importance of the predictor in the final model, calculated as the proportion of top models the predictor was included in.

^d^ Genome-wide heterozygosity (*H*_GW_) and MHC heterozygosity (*H*_MHC_) were standardised across all loci for which an individual was genotyped to reduce the influence of missing data on the analysis.

Supplementary Table 5: Top model set (top 2 AIC_C_) of number of offspring models, using overall and relative (standardised across competitive breeding enclosure) measures.

Female models were fitted as generalised linear mixed models with a random intercept for “enclosure.year” and a two-column binomial response of successes/failures. Male models were fitted as generalised linear models with a Poisson distribution. The final models are provided in Supplementary Tables 3 and 4.

| Model statement | AIC_C_ | Δ*_i_*^a^ | *w_i_*^b^ | R^2 c^ |
| --- | --- | --- | --- | --- |
| *Females (overall)* |  |  |  | 0.116 |
| β_0_ + *H*_MHC_^d^ + Average weight | 104.7 |  | 0.248 | 0.114 |
| β_0_ + Average weight | 105.6 | 0.88 | 0.159 | 0.148 |
| β_0_ + *H*_GW_^d^ + Average weight | 106.3 | 1.61 | 0.111 | 0.164 |
| β_0_ + *H*_MHC_^d^ + *H*_GW_^d^ + Average weight | 106.3 | 1.63 | 0.110 | 0.121 |
| *Males (overall)* |  |  |  | 0.352 |
| β_0_ | 99.3 |  | 0.131 | 0 |
| β_0_ + Average weight | 99.3 | 0.08 | 0.126 | 0.133 |
| β_0_ + *H*_MHC_^d^ | 100.1 | 0.84 | 0.086 | 0.090 |
| β_0_ + Age | 100.1 | 0.89 | 0.084 | 0.087 |
| β_0_ + *H*_MHC_^d^ + *H*_GW_^d^ | 100.3 | 1.02 | 0.078 | 0.221 |
| β_0_ + Age + Average weight | 100.4 | 1.11 | 0.075 | 0.217 |
| β_0_ + *H*_MHC_^d^ + Average weight | 100.5 | 1.19 | 0.072 | 0.212 |
| β_0_ + *H*_GW_^d^ | 100.6 | 1.33 | 0.068 | 0.062 |
| *Females (relative)* ^e^ |  |  |  | 0.158 |
| β_0_ + z.Average weight | 99.8 |  | 0.413 | 0.139 |
| β_0_ + z.*H*_MHC_^d^ + z.Average weight | 101.6 | 1.81 | 0.167 | 0.150 |
| *Males (relative)* ^e^ |  |  |  | 0.399 |
| β_0_ + z.*H*_GW_^d^ | 98.5 |  | 0.154 | 0.177 |
| β_0_ + z.*H*_MHC_^d^ + z.*H*_GW_^d^ | 99.1 | 0.51 | 0.119 | 0.284 |
| β_0_ | 99.3 | 0.72 | 0.108 | 0 |
| β_0_ + z.*H*_GW_^d^ + z.Average weight | 99.6 | 1.01 | 0.093 | 0.259 |
| β_0_ + z.Average weight | 99.6 | 1.09 | 0.089 | 0.116 |
| β_0_ + z.Age + z.*H*_GW_^d^ | 99.6 | 1.09 | 0.089 | 0.255 |
| β_0_ + z.*H*_MHC_^d^ + z.*H*_GW_^d^ + z.Average weight | 100.4 | 1.84 | 0.061 | 0.356 |

^a^ Change in AIC_C_ from the best model.

^b^ Akaike model weight.

^c^ For female models, conditional R^2^ calculated for global model as Nakagawa & Schielzeth (2013), and then separately for each of the submodels in the top model set. For male models, the pseudo-R^2^ (Nagelkerke, 1991) is reported.

^d^ Genome-wide heterozygosity (*H*_GW_) and MHC heterozygosity (*H*_MHC_) were standardised across all loci for which an individual was genotyped to reduce the influence of missing data on the analysis.

^e^ All predictors were converted to z-scores within each enclosure year and sex before input to models to reflect competition amongst individuals.

**Supplementary Figure 1: Scatterplots of the relationship between *H*_GW_ and *H*_MHC_ for females and males.**

Correlation coefficients are -0.21 (SE = 0.15) for females, and -0.09 (SE = 0.15) for males.


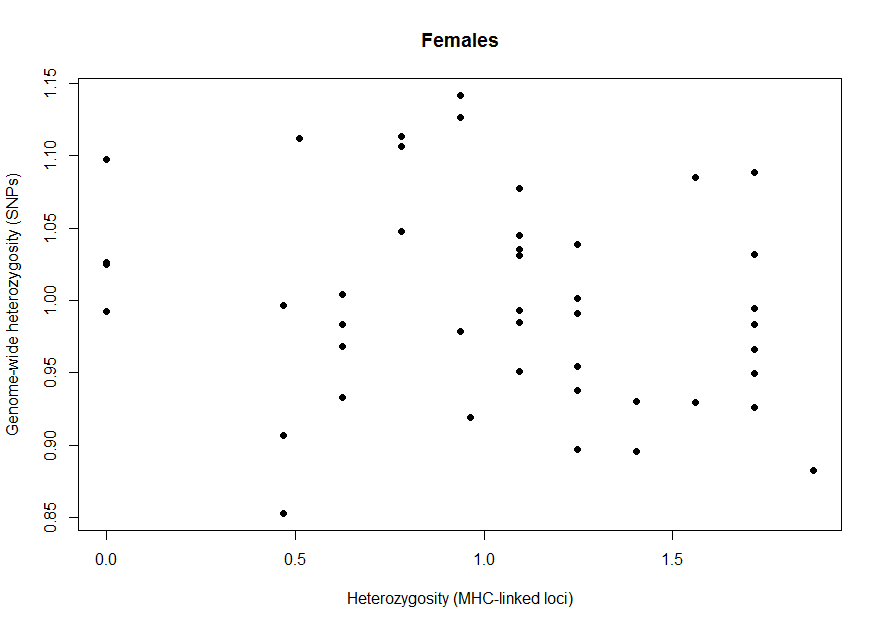


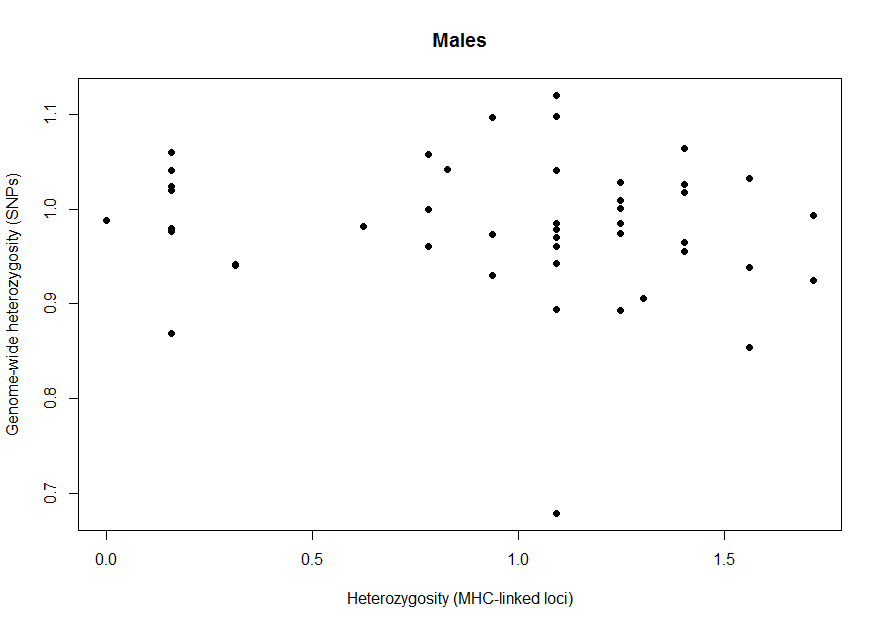


Literature Cited in Supplementary Material

Cheng, Y. & Belov, K. (2014) Characterisation of non-classical MHC class I genes in the Tasmanian devil (*Sarcophilus harrisii*). *Immunogenetics*, 66, 727-735.

Day, J., Gooley, R.M., Hogg, C.J., Belov, K., Whittington, C.M. & Grueber, C.E. (2019) MHC- associated mate choice in captive versus wild Tasmanian devils. *Behavioral Ecology*, arz092.

Gelman, A. (2008) Scaling regression inputs by dividing by two standard deviations. *Statistics in Medicine*, 27, 2865-2873.

Nagelkerke, N.J.D. (1991) A note on a general definition of the coefficient of determination. *Biometrika*, 78, 691-692.

Nakagawa, S. & Schielzeth, H. (2013) A general and simple method for obtaining R2 from generalized linear mixed effect models. *Methods in Ecology and Evolution*, 4, 133-142.
